# Saturation sampling for spatial variation in multiple air pollutants across an inversion-prone metropolitan area of complex terrain

**DOI:** 10.1186/1476-069X-13-28

**Published:** 2014-04-16

**Authors:** Jessie LC Shmool, Drew R Michanowicz, Leah Cambal, Brett Tunno, Jeffery Howell, Sara Gillooly, Courtney Roper, Sheila Tripathy, Lauren G Chubb, Holger M Eisl, John E Gorczynski, Fernando E Holguin, Kyra Naumoff Shields, Jane E Clougherty

**Affiliations:** 1 Department of Environmental and Occupational Health, University of Pittsburgh Graduate School of Public Health, Pittsburgh, PA, USA; 2 E & G Environmental Diagnostics, LLC, Warwick, NY, USA; 3 Department of Medicine, Division of Pulmonary, Allergy, and Critical Care Medicine, University of Pittsburgh, Pittsburgh, PA, USA

**Keywords:** Air pollution monitoring, Black carbon (BC), Fine particulate matter (PM_2.5_), Airborne particles with a diameter of 2.5 micrometers or less, Geographic information systems (GIS), Nitrogen dioxide (NO_2_), Spatial variability, Temperature inversion

## Abstract

**Background:**

Characterizing intra-urban variation in air quality is important for epidemiological investigation of health outcomes and disparities. To date, however, few studies have been designed to capture spatial variation during select hours of the day, or to examine the roles of meteorology and complex terrain in shaping intra-urban exposure gradients.

**Methods:**

We designed a spatial saturation monitoring study to target local air pollution sources, and to understand the role of topography and temperature inversions on fine-scale pollution variation by systematically allocating sampling locations across gradients in key local emissions sources (vehicle traffic, industrial facilities) and topography (elevation) in the Pittsburgh area. Street-level integrated samples of fine particulate matter (PM_2.5_), black carbon (BC), nitrogen dioxide (NO_2_), sulfur dioxide (SO_2_), and ozone (O_3_) were collected during morning rush and probable inversion hours (6-11 AM), during summer and winter. We hypothesized that pollution concentrations would be: 1) higher under inversion conditions, 2) exacerbated in lower-elevation areas, and 3) vary by season.

**Results:**

During July - August 2011 and January - March 2012, we observed wide spatial and seasonal variability in pollution concentrations, exceeding the range measured at regulatory monitors. We identified elevated concentrations of multiple pollutants at lower-elevation sites, and a positive association between inversion frequency and NO_2_ concentration. We examined temporal adjustment methods for deriving seasonal concentration estimates, and found that the appropriate reference temporal trend differs between pollutants.

**Conclusions:**

Our time-stratified spatial saturation approach found some evidence for modification of inversion-concentration relationships by topography, and provided useful insights for refining and interpreting GIS-based pollution source indicators for Land Use Regression modeling.

## Background

Advances in intra-urban air monitoring, such as spatial saturation sampling and land use regression (LUR) modeling [1-8], have substantially improved epidemiological estimation of air pollution impacts on health in urban areas [9,10]. However, few studies have been designed to capture spatial variation during select hours of the day, and important challenges remain for incorporating time-varying meteorological factors and local topography into the assessment of fine-scale spatial variation in air quality [11,12]. The Pittsburgh metropolitan area represents an opportunity to extend air monitoring methods to address spatial and temporal drivers of air quality variability – specifically spatial confounding among multiple pollution sources (e.g., legacy industry, vehicle traffic), and potential modifiers of source-concentration relationships (e.g., elevation, temperature inversions) – toward better characterizing risk factors for multiple health outcomes, and growing regional health disparities [13,14].

Spatial saturation monitoring and land use regression (LUR) modeling are standard exposure assessment methodologies for characterizing intra-urban variability in air pollution concentrations [1,4-6,11] and pollution source apportionment [15]. For spatial saturation studies, Geographic Information System (GIS)-based indicators of local air pollution sources are used to systematically allocate monitoring locations to saturate hypothesized pollution concentration gradients across complex domains. This spatially-informed study design enables estimation of fine-scale variation in air quality, and can generate chronic air pollution exposure assessments for environmental epidemiology.

Integrating primarily spatial (e.g., distribution of roadways and industrial facilities) and temporal (e.g., temperature, weather) factors that contribute to local air pollution, and understanding their potential interactions, is an important methodological challenge for LUR-based analysis. One common approach to disentangling spatial and temporal factors is designating ‘reference,’ or ‘background,’ monitors to, first, determine the proportion of local pollution explained by temporally-varying factors (e.g., meteorology, long-range transport) [7,16], and, second, to adjust samples collected at different locations and points in time to indicate seasonally-representative concentrations. As such, siting reference monitors is important for robust study design; it is well established that locating monitors away from local source influence will produce more accurate measurements of temporally-mediated pollution patterns [17], but there is little guidance in the literature to help investigators target meteorological interactions with local topography, especially for chemically reactive or photochemically active pollutants (e.g., NO_2_). As more urban studies are monitoring multiple pollutants, for which the relative spatio-temporal components are different [18], the citing of reference monitors in a way that is interpretable for multiple pollutants and across topographic regimes is an important challenge.

Despite over two decades of air quality improvements following the decline of the steel industry in western Pennsylvania, the Pittsburgh metropolitan area exceeds national health-based air quality standards for criteria pollutants [19]. While high air pollution levels are partially attributable to regional transport of emissions from coal-fired power plants of the Ohio valley, local pollution sources are substantial drivers [20-22]. Local emissions inventories are dominated by a small number of remaining large industrial facilities strategically located along river valleys [19], including the nation’s largest coke works, which sits approximately 24 kilometers south of downtown. A diverse transportation network of rail, barge, diesel trucks, and passenger vehicles contribute mobile emissions. Though a relatively small city (approximately 300,000 residents), urban sprawl and roadway vehicle congestion is a substantial problem, as a large number of tunnels and bridges lead to traffic bottlenecks, and some highway segments rank among the twenty most congested outside of Los Angeles and New York City [23]. Finally, population susceptibility factors (e.g., poor access to healthcare, concentrated poverty) are spatially patterned with topography and pollution sources, clustered in industrial river valleys, creating the potential for joint and synergistic health effects. In the City of Pittsburgh, for example, median household income is approximately $10,000 less among census block groups in the 20^th^ percentile of elevation, as compared to highest-elevation (80^th^ percentile) census block groups [24].

In the Pittsburgh metropolitan area, complex topography at the confluence of three rivers combines with meteorology to drive frequent atmospheric temperature inversions [25], which can prevent vertical dispersion of airborne pollutants, trapping emissions near the earth’s surface. Inversion layers form when the warm-to-cool vertical temperature gradient of the atmosphere is inverted, and are generally accompanied by low surface wind speeds. Causes of inversions are dependent upon local topography and meteorology interactions, including when rapid overnight cooling of the earth’s surface causes cooling of air near the surface, compared to higher altitude air (i.e., radiative inversion), or when high pressure systems descend into relatively cooler low elevation areas (i.e., subsidence), both of which may occur in the Pittsburgh region. In areas of complex terrain, inversion effects on local air pollution may be exacerbated in low-lying areas and valleys, where the earth’s surface is shadowed and slower to warm with the sunrise [26,27]. Inversions have been linked with acute pollution events of photochemical smog and particulate matter, such as occurred in nearby Donora, PA in 1948 [28], and have been linked with cardiovascular [29] and respiratory health [30,31]. In the pilot mobile monitoring study which informed the design of this campaign, we identified regional inversion conditions on 50% of summertime sampling days, and, accordingly, PM_2.5_ concentrations were greater during morning sampling hours (8-10 AM) than afternoon (12-2 PM) [32].

Here, we present a spatial saturation approach designed to capture the impact of inversion effects across complex terrain – towards evaluating the efficacy of topographic information for capturing potential modification of local source-concentration relationships by meteorology. We describe study design and implementation of a two-season (winter and summer) multi-pollutant monitoring campaign across the Pittsburgh metropolitan area, using programmable integrated monitors to sample solely during morning hours when atmospheric inversions are most frequent. We report GIS-based methods for systematically allocating monitors across locally-specific pollution source and topography profiles, and address challenges of distinguishing spatial and temporal components of local pollution variation in different pollutants by comparing two temporal adjustment approaches. This is the first study, to our knowledge, to explicitly capture spatial variation in pollution during selected hours of the day – here, targeting topography-meteorology interactions by collecting spatially-distributed samples only during inversion-prone hours of the day, and including topography in monitoring site allocation. Air quality data derived from this study will ultimately be applied towards: (a) LUR modeling of intra-urban variability in multiple pollutants and seasons, and (b) epidemiologic investigation of health outcomes.

## Methods

We used GIS-based indicators of local pollution sources and topography to systematically allocate 36 air monitoring sites across the metropolitan area during two seasons – June-August 2011 (summer) and January-March 2012 (winter). The same sites were repeated in each season, within which monitors were distributed between six 5-day weekday sessions in each season. We collected integrated samples of criteria pollutants, and derived seasonal averages using two reference monitors – one urban and one regional background. We tested the hypothesis that lower-elevation areas may experience higher pollution concentrations under inversion conditions [20,32], and that these effects may vary by season.

### Sampling instrumentation and laboratory analyses

We collected integrated samples of nitrogen dioxide (NO_2_), ozone (O_3_), sulfur dioxide (SO_2_), fine particulate matter (PM_2.5_) black carbon (BC), and constituents using portable ambient air sampling units originally designed for the New York City Community Air Survey [7]. Particle sampling instruments include a Dual Stage PM_2.5_ Harvard Impactor (Air Diagnostics and Engineering Inc.) with particulate matter collected onto 37 mm Teflon filters (PTFE membrane, 2 μm pores, Pall Life Sciences), a HOBO data logger for relative humidity, temperature, and barometric pressure readings (Onset Computer Corporation). Battery-operated vacuum pumps (SKC, Inc.) moved ambient air through particle filters at a constant rate of 4 liters per minute, and pre - and post-flow rates were recorded for data quality assurance. Passive gaseous samplers (Ogawa & Co. USA) were placed into weather tight shelters on the exterior of sampling units. Sampling instruments were housed in weather tight boxes, and mounted 3-4 meters above ground on utility poles, near the breathing zone.

PM_2.5_ and BC were measured solely during weekday morning rush hours and potential inversion hours, using a chrontroller (ChronTrol Corporation) to program the sampling units to simultaneously sample all locations (including reference sites) each weekday (Monday-Friday) from 6:00 AM to 11:00 AM. Deployment and retrieval schedules were aimed at minimizing differences in exposed time for passive badges between monitors and across sessions.

Teflon filters were pre- and post-weighed at the University of Pittsburgh, Department of Environmental & Occupational Health, in a temperature and relative humidity-controlled glove box (PlasLabs Model 890 THC) using an ultra-microbalance (Mettler Toledo Model XP2U) for total PM_2.5_ mass, and reflectometry for BC absorbance was performed using the EEL43M Smokestain Reflectometer (Diffusion Systems). Ogawa passive badges were analyzed at the University of Pittsburgh, Department of Geology & Planetary Sciences using water-based extraction and spectrophotometry (Thermo Scientific Evolution 60S UV-Visible Spectrophotometer) for NO_2_ ppb concentration. SO_2_ and O_3_ sample analyses are ongoing, and we do not report their results here.

### Quality assurance and controls

To account for possible contamination, we used one laboratory blank and multiple field blanks each session for gases and particles, and co-located paired distributed monitors at four randomly-selected sites during one sampling session each season. PM_2.5_ pump flow rates were calibrated to 4.0 liters per minute (LPM) (temperature-adjusted based on weather forecasts) prior to deployment, and compared to post-collection rates. We verified program completion for each sampler run using the sampling unit program log.

Summer sampling was performed from July 25 to September 9, 2011 (the week of August 29 skipped for logistical reasons), and winter sampling from January 16 to February 24, 2012. Across seasons, all PM_2.5_ samples met acceptable pre- and post-collection flow rate (within 5% of 4.0 LPM). Instrumentation failure occurred at only one site, which was re-sampled during a later session. Co-located measures of PM_2.5_ and NO_2_ were highly correlated (rho = 0.93 and 0.97, respectively) across four monitoring locations. Field blanks for PM_2.5_ and NO_2_ ranged from 0.07-1.50 μg/m^3^ and 0.01-0.05 ppb, respectively, and were similar across seasons. Pollutant concentrations were field blank-corrected. Data completeness was 100% for PM_2.5_, NO_2_, and BC, with no statistical outliers (outside of mean +/- 3 standard deviations).

### Study domain selection and characterization

**Figure 1:**
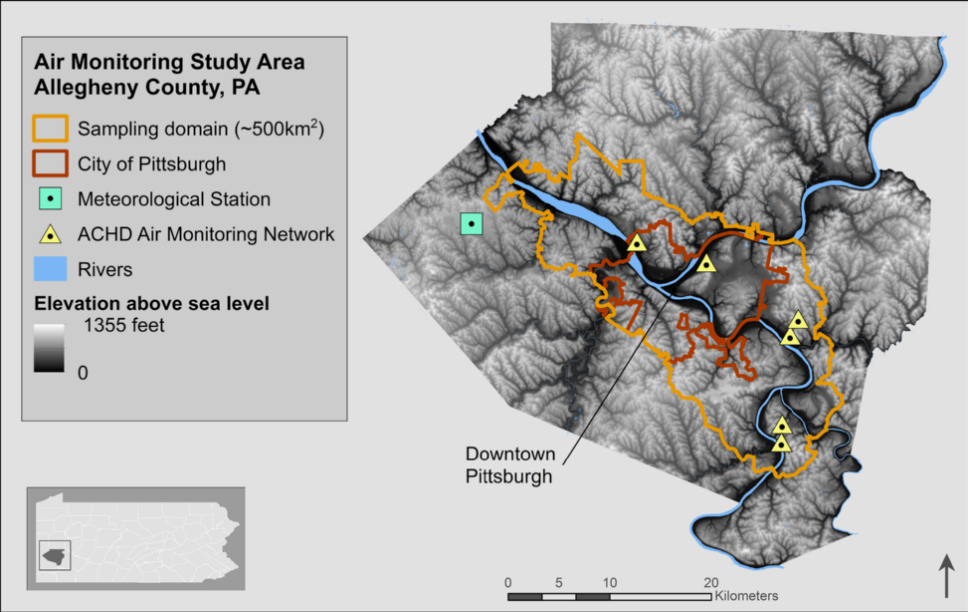
Air monitoring study domain.

We aimed to capture large industrial point sources, major roadways, and river valleys across an urban-to-suburban gradient of Allegheny County, within a feasible coverage area, extending at least 10 km Northeast of industrial point sources, with respect to the prevailing wind direction (West/Southwest). In a GIS, we fit a polygon to meet coverage and distance criteria, and selected intersecting contiguous census tracts, to enable subsequent merging of population indicators. Our domain stretched northwest of downtown Pittsburgh along the Ohio River, and southeast along the Monongahela River, covering approximately 500 km^2^, including 258 contiguous census tracts within Allegheny County, PA (Figure 1), and captured wide variability in population density: from 272 to 55,343 residents per km^2^[24]. Large industrial point sources within our domain include two coke smelting works (Neville Island and Clairton) and a steel mill (Braddock).

For purposes of sampling site selection, we explored spatial variability across a range of local source indicators, and potential modifiers of source-concentration relationships. Based on recent source apportionment of PM_2.5_ measurements collected at Allegheny County Health Department (ACHD) regulatory monitors, which attributed the majority of measured fine particles to local industrial and mobile sources [19], we developed GIS-based indicators of local industrial emissions and on-road vehicle traffic. Because traffic-related pollution varies within 50-200 m from roadways [33,34], and because of steep elevation gradients in the Pittsburgh area, we used relatively small regular 100 m^2^ lattice grid cells to characterize the study domain according to three key local pollution indicators: (a) traffic density, (b) emission-weighted proximity to industrial point sources, and (c) topography. GIS-based analysis and mapping were implemented in ArcInfo, v10 (ESRI, Redlands, CA).

We evaluated multiple indicators of traffic emissions (e.g., proximity to roadways, heavy track traffic), and decided on the most inclusive indictor – total on-road traffic density – to prevent biasing our study design toward one class of vehicle emissions. First, we created road-segment counts by summing total vehicles on major road segments plus an estimated 500-vehicle count on minor road segments (based on major road count distribution), using Pennsylvania Department of Transportation Annualized Average Daily Traffic (AADT) counts (2011) [35]. Using ArcInfo’s Spatial Analyst toolbox, we derived a continuous kernel traffic density surface by applying a Gaussian decay function to traffic counts on all road segments within our domain. From this traffic density surface, we calculated mean traffic density within each 100 m^2^ grid cell.

We created a multi-pollutant indicator of industrial emissions to prevent biasing our sampling design toward one pollutant or industry type. Using emissions data from the U.S. Environmental Protection Agency’s National Emissions Inventory [36], we first summed emissions mass in tons of multiple pollutants PM_2.5_ (filterable and condensable), nitrogen oxides (NO_X_)_,_ sulfur dioxide (SO_2_), and volatile organic compounds (VOCs) – from reporting facilities in Allegheny County, PA. We then used inverse-distance interpolation to calculate an emission-weighted proximity to industry indicator for each 100 m^2^ grid cell centroid, drawing emissions information from facilities within an 80 km radial buffer threshold. Inverse-distance interpolation weights emissions values at locations in between facilities as a function of distance, such that relatively near facilities will have a greater influence than far facilities on local air quality.

As there is no standard metric to demarcate ‘valley’ versus ‘non-valley’ areas, we opted to use continuous elevation above sea level to maximize spatial resolution and comparability with previous LUR studies [8,37-39]. We calculated mean elevation within each 100 m^2^ grid cell from the U.S. Geological Survey National Elevation Dataset 30 m^2^-resolution raster data set [40]. Across sampling locations, elevation is correlated with distance-to-river-centerlines at rho = 0.67, supporting our interpretation of elevation as an indicator of river valleys, where cool air pools may exacerbate inversion formation. Furthermore, in our pilot mobile monitoring study, we found a strong relationship between elevation, atmospheric inversions, and PM_2.5_ and PM_10_ concentrations in one relatively low-lying Pittsburgh community (Braddock, PA) [32].

### Distributed site selection & allocation

**Figure 2:**
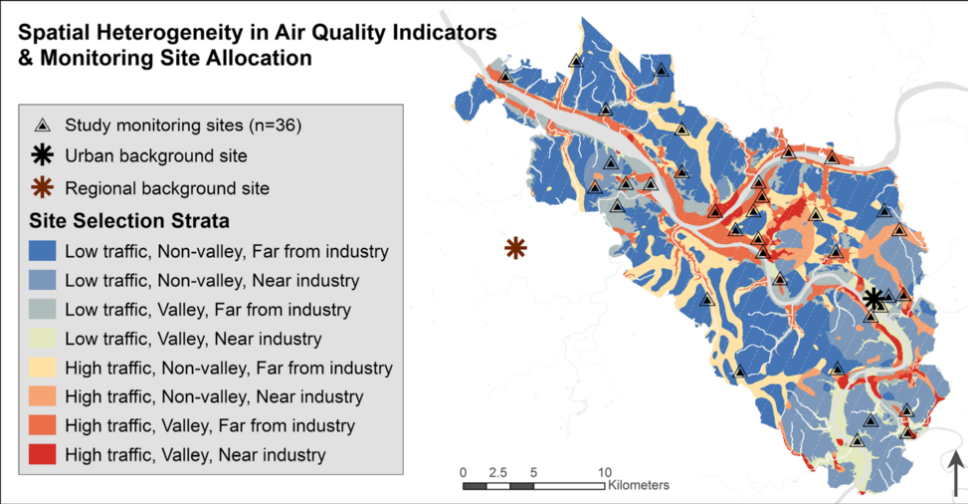
Pollution monitoring locations across source indicators and inversion-prone areas.

Across our study domain, the distribution of source indicators used for sampling site selection – traffic density, emission-weighted proximity to industrial facilities, and elevation – varied substantially (Figure 2); source indicators were not collinear (rho = -0.08 to -0.21, across all 100 m^2^ grid cells). We dichotomized each source indicator at the 70^th^ percentile, and cross-stratified each 100 m^2^ grid cell across eight classifications, representing combinations of ‘high’ and ‘low’ source profiles (e.g., ‘low’ traffic density, ‘near’ industrial sources, low elevation ‘valley’). This dichotomization point was chosen based on left-skewed distribution of source indicators, to systematically over-sample hypothesized high-pollution areas.

We used stratified random sampling without replacement to select 30 spatially-distributed monitoring grid cells across eight source indicator cross-strata, using Geospatial Modeling Environment software, v 0.7.2.0 (Spatial Ecology, LLC). Six additional grid cells were selected to fill spatial gaps in the periphery of our large domain. Specifically, three 30 km^2^ areas in which no cells had been allocated were selected in GIS, and two cells randomly selected from each. Rivers and riverbank areas (<20 m from a river’s edge) were not eligible for sampling site selection, for logistical reasons. Sample size was determined by available resources, domain size, logistical limitations, and precedent of 40 monitoring sites for urban LUR modeling [41,42]. Figure 2 shows spatial allocation of distributed and reference monitoring sites, which were repeated in summer and winter.

Suitable locations to mount sampling units (e.g., utility or telephone poles) were identified near the centroid of selected 100 m^2^ grid cells by field teams, using consistent protocols. Mounting pole eligibility criteria included: no obstructions within 3 m of the monitors, street accessible, three or more meters from buildings, identifiable pole ownership (to obtain permissions), away from bus stops, and without overhanging tree branches. Latitude and longitude coordinates of selected mounting poles were pinpointed using GPS (Colorado^®^ 400 t, Garmin), and verified in Google Earth™. A detailed site survey was conducted for each sampling location, to document relevant information potentially unavailable in GIS datasets (e.g., construction). Permissions to mount monitors on utility poles were obtained from Duquesne Light Co., Verizon, Inc., Allegheny County Parks Department, and the City of Pittsburgh Department of Public Works.

As sampling at the 36 sites was evenly allocated across six Monday-through-Friday sampling sessions (six sites sampled per session), we sought to balance source indicator strata and spatial distribution across sessions to avoid confounding spatial and temporal patterns in pollution concentrations. For each session, we used traffic density, the most spatially dispersed indicator, to draw a stratified-random sample (without replacement) of six sites (e.g., randomly allocate 3 ‘high’ and ‘low’ traffic density sites per session). Because pollution source and topography indicators may be spatially clustered in Pittsburgh (i.e., industrial facilities located in low-elevation river valleys and/or near highways), we required spatial representation of four regions of our domain (i.e., east and west banks of the Monongahela River, northeast and southeast of downtown) within each session. Temporal allocation of sites across sessions was the same during winter and summer sampling seasons.

### Reference monitors and temporal adjustment

**Figure 3:**
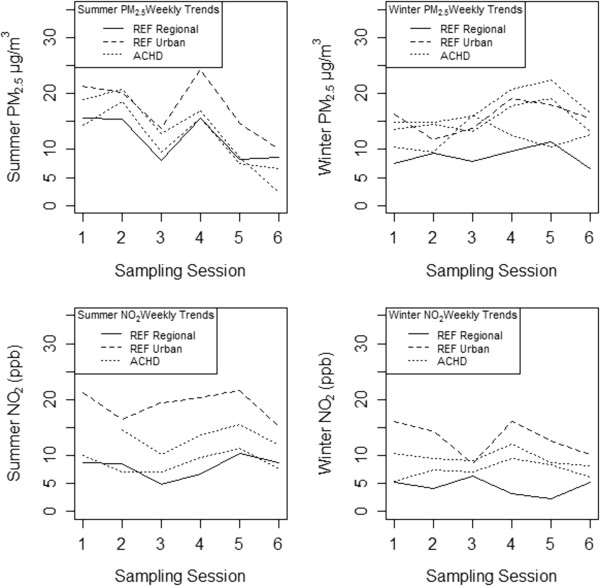
**Weekly PM**_**2.5 **_**and NO**_**2 **_**trends between study reference sites and ACHD regulatory monitors, by season.**

We designated two reference sites, which were sampled during all sessions to provide information on overall temporal trends in air quality. First, an upwind reference site (Regional background site – Figure 2) located in a relatively rural area west of our domain, in Settlers Cabin County Park, Oakdale, PA, would provide information on regional background air quality. Second, a relatively urban reference site (Urban background site – Figure 2) within our domain, in Braddock, PA, was selected for comparison. The urban reference site is located in a low-elevation area, to capture topography-related inversion effects in seasonal air quality trends. We compared ACHD regulatory monitoring data to the weekly temporal patterns in NO_2_ and PM_2.5_ measured at study reference monitors, and found variable correlation between both reference monitors with ACHD monitors (Spearman rho from -0.71 to 0.90 (mean = 0.23)). Figure 3 plots weekly PM_2.5_ and NO_2_, trends across ACHD regulatory monitors and study reference monitors (regional and urban background); regional and urban reference trends are variably correlated in both seasons (Spearman rho 0.04 to 0.91). As expected, regional background concentrations were consistently lower than urban reference site measurements, and lower than ACHD regulatory monitors. This difference is larger for NO_2_ in both seasons, compared to PM_2.5_.

To facilitate comparison between site-specific concentrations, collected during one of six sampling sessions, we apply a temporal adjustment to adjust distributed site samples for between-session variability primarily driven by time-varying meteorology or long-range transport, and to derive seasonally representative mean values. Specifically, to estimate the expected, seasonally representative concentration at a given site – as if it had been sampled during an “average” week – the observed concentration is multiplied by the ratio of the seasonal average reference concentration, and then divided by the session-specific reference concentration. As such, it is the relationship of the session relative to the seasonal average at the reference site(s) that determines the temporal adjustment, which can therefore adjust distributed concentration to both lesser and greater values. These adjusted seasonal mean values allow for examination of spatial source-concentration relationships, with reduced influence of time-varying factors (i.e., meteorology, long-range transport). Because the appropriate reference trend for temporal adjustment may vary by season and/or pollutant, we evaluate two methods: one using the only the regional background reference trend (Equation 1), and a second using the mean trend of the urban and regional background sites (Equation 2). Both of these approaches have been successfully applied in other studies of intra-urban air quality variability [7,16].

Within each season, sampled pollutant concentrations were temporally adjusted as:

(1) $${\left[\text{adjConc}\right]}_{\mathrm{ij}}=\frac{{\left[\text{Conc}\right]}_{\mathrm{ij}}}{{\left[{\text{Ref}}_{\text{Regional}}\right]}_{j}}*{\left[{\text{Ref}}_{\text{Regional}}\right]}_{\text{Season}}$$

(2) $${\left[\text{adjConc}\right]}_{\mathrm{ij}}=\frac{{\left[\text{Conc}\right]}_{\mathrm{ij}}}{{\left[{\text{Ref}}_{\mu \left(\text{Regional}+\text{Urban}\right)}\right]}_{j}}*{\left[{\text{Ref}}_{\mu \left(\text{Regional}+\text{Urban}\right)}\right]}_{\text{Season}}$$

where [adjConc]_ij_ is the temporally-adjusted pollutant concentration at monitoring site i during sampling session j, [Conc]_ij_ is the pollutant concentration at monitoring site i during sampling session j, [Ref_Regional_]_j_ is the regional background reference site concentration during sampling session j, [Ref_*μ*(Regional+Urban)_]_j_ is the mean concentration of the regional background and urban reference sites during sampling session j, [Ref_Regional_]_Season_ is the seasonal average regional background reference site pollutant concentration, and [Ref_*μ*(Regional+Urban)_]_Season_ is the mean seasonal average pollutant concentration of the regional background and urban reference sites.

### Temperature inversions and meteorology

We identified probable morning inversion hours as 6:00-11:00 AM by examining: (a) meteorological sounding data, (b) hourly ACHD regulatory monitor data, and (c) pilot mobile monitoring study data [32]. We used meteorological sounding data (i.e., Skew-T diagrams) recorded daily at 7:00 AM from the Pittsburgh International Airport, approximately 25 km Northwest of downtown Pittsburgh (Figure 1), to identify lapses in the vertical temperature gradient characteristic of inversion events. To confirm the number of inversion hours overlapping with sampling intervals (6:00-11:00 AM), inversion hours per event were evaluated using Bufkit 10.11, a forecast profile visualization and analysis software developed by the National Oceanic and Atmospheric Association (NOAA) and National Weather Service [32]. Inversions were defined as two or more hours of inverted temperature gradient during sampling hours. Inversion frequency was operationalized as number of inversion mornings per sampling session (1-4), and as a binary indicator (fewer than 3, vs. 3 or more days per session), based on overall frequency distribution. Importantly, these characterizations are regional scale, and do not reflect the complex interactions between topography, surface thermal variability in urbanized areas (i.e., urban heat island effect), and pollution.

Wind speed and direction influence local pollution concentrations through horizontal advection, however, the metrics that can elucidate spatial gradients in these processes are not well specified [43]. Wind speed and direction data measured at NOAA’s weather station at the Pittsburgh International Airport (and obtained from NOAA’s online National Climatic Data Center) were clipped to each sampling session, and used to generate wind rose diagrams (using Lakes Environmental WRPLOT View freeware) to examine within and between session variability. We then determined dominant wind direction and average wind speed (from any direction) for each sampling session. We compared wind speed and direction on inversion versus non-inversion mornings, in each season, to better understand the relationship between inversion conditions and local pollutant concentrations.

### Statistical analysis

We calculated descriptive statistics for PM_2.5_, BC and NO_2_, during each season, to identify potential outliers, and to compare temporally adjusted values by method (i.e., Regional-only vs. Urban + Regional). We examined pollutant concentration distributions across pollution indicator strata used for site selection and allocation: traffic density, emission-weighted proximity to industry, and elevation above sea level using Spearman correlation analysis, to account for non-normal distribution of pollution concentrations. We examined between-season differences using paired t-tests on log-transformed (base 10) concentrations, to account for non-normality of distributions, and compared results across temporal adjustment methods. We examined the relationship between log-transformed pollutant concentrations and inversion frequency, by elevation and temporal adjustment method. Further analyses of meteorological factors examined associations between temporally adjusted pollutant concentrations and within-session average wind speed (continuous and binary (median-stratified) measures), and dominant wind direction (e.g., West, Northwest). Statistical analyses were performed in SAS, v 9.2 (Cary, NC) and R statistical software v 2.12.1.

## Results

PM_2.5_, BC, and NO_2_ concentrations varied across monitoring locations, capturing a wider range of concentrations than at ACHD regulatory monitoring locations during corresponding sampling weeks. Table 1 reports summary statistics of pollutant concentrations measured across distributed sites, by season, and compares temporal adjustment methods (Equations 1 and 2). Under both temporal adjustment methods, NO_2_ concentrations were higher during winter (p < 0.001), and PM_2.5_ concentrations were higher during summer sampling (p < 0.10). Within-season, distributed pollution measurements varied by temporal adjustment method, (e.g., summer NO_2_ under regional-only vs. urban and regional background adjustment), with Spearman rho values ranging from 0.59 to 0.95. Specifically, adjustment using the mean of urban and regional background trends (Equation 2) produced attenuated seasonal average concentrations across sites, particularly for NO_2_, in both seasons (Table 1). Weakest between-method correlations were observed for summer NO_2_ and winter PM_2.5_ (rho 0.59 and 0.60, respectively), and strongest correlation for BC in both seasons (summer rho 0.81, winter 0.95).

**Table 1:** **Summary statistics of PM**_**2.5 **_**(μg/m**^**3**^**), BC (abs), and NO**_**2 **_**(ppb) concentrations, by season, comparing temporal adjustment methods**

	**Urban and Background Adjustment (n = 36)**				**Background-only Temporal Adjustment (n = 36)**			
	**Mean**	**SD**	**Minimum**	**Maximum**	**Mean**	**SD**	**Minimum**	**Maximum**
Summer PM_2.5_ (μg/m^3^)	14.00	3.68	1.28	21.06	14.24	3.97	1.33	22.71
Summer BC (abs)	1.58	0.85	0.02	4.82	1.61	0.91	0.02	4.64
Summer NO_2_ (ppb)	10.75	3.36	5.12	17.25	12.30	6.50	4.46	27.72
Winter PM_2.5_ (μg/m^3^)	12.53	2.28	8.28	16.98	12.64	2.52	8.02	20.10
Winter BC (abs)	1.31	0.55	0.63	2.99	1.31	0.53	0.70	2.72
Winter NO_2_ (ppb)	17.93	3.90	9.93	25.60	18.69	6.21	10.90	34.10

**Figure 4:**
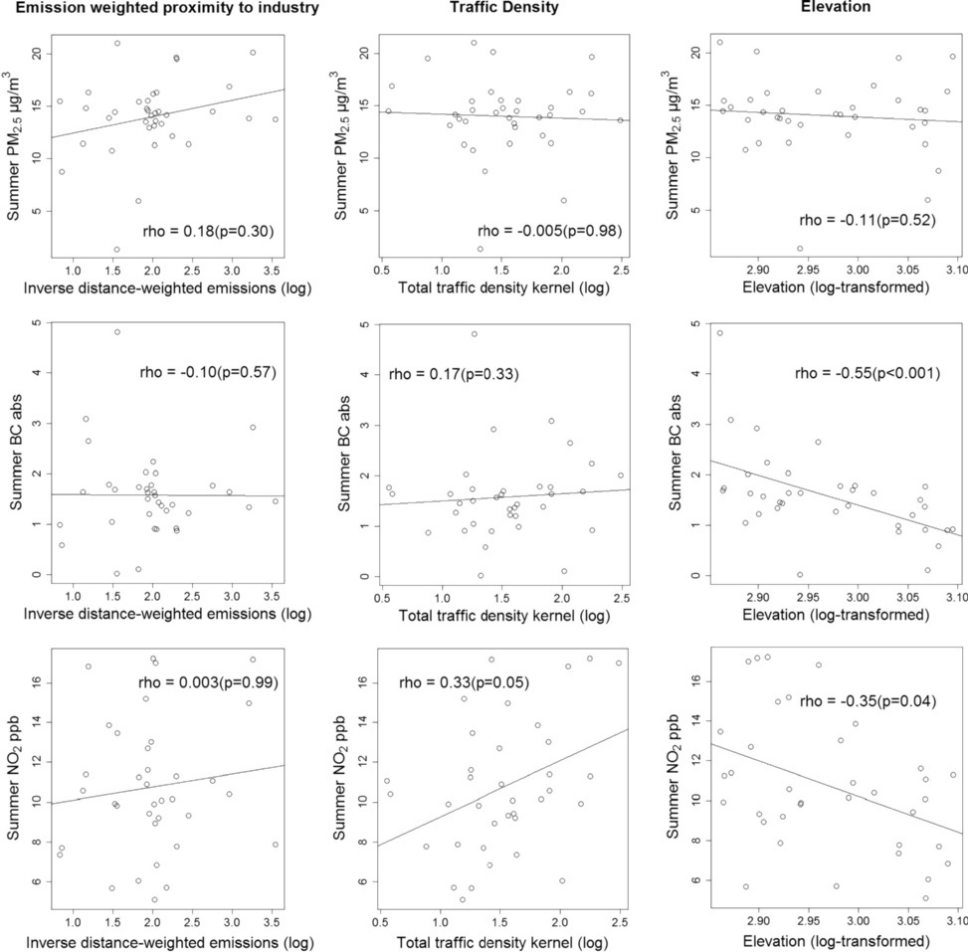
**Summer PM**_**2.5**_**, BC and NO**_**2 **_**concentrations across source indicators and elevation (temporally adjusted using regional and urban background reference trends).**

**Figure 5:**
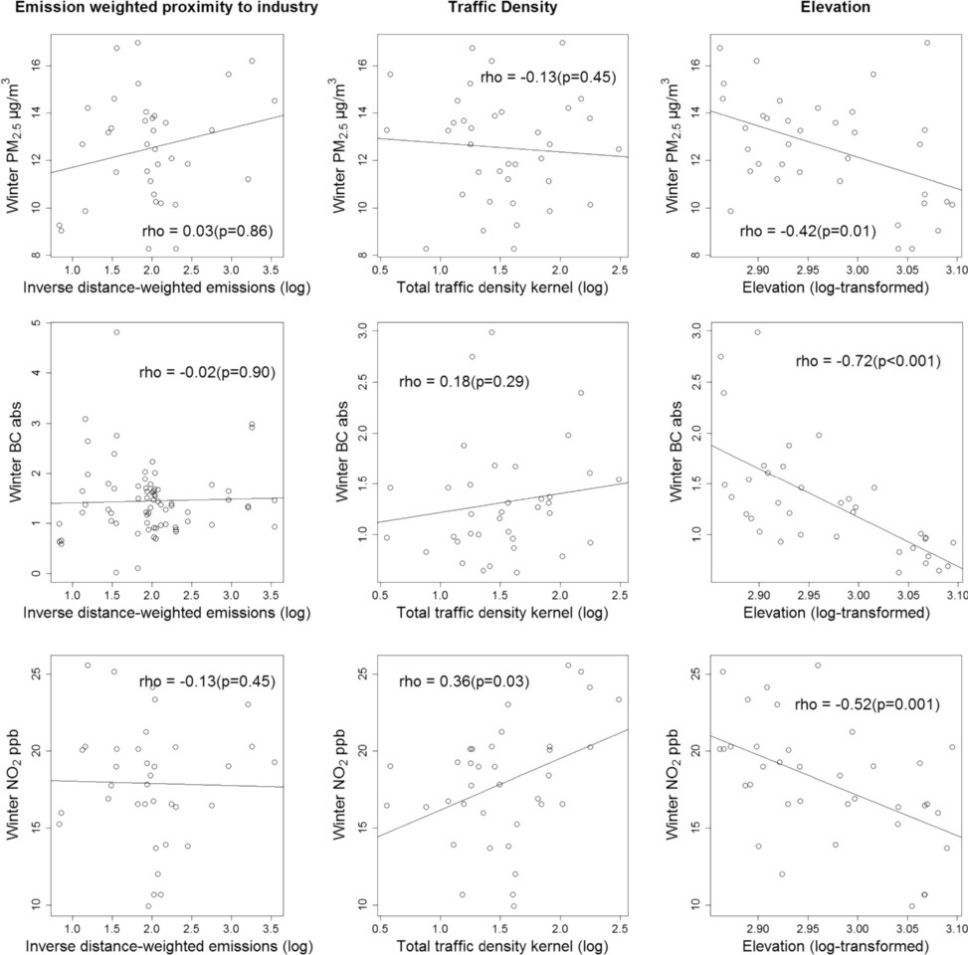
Winter pollutant concentrations across source indicators and elevation (temporally adjusted using regional and urban background reference trends).

Figures 4 and 5 show scatterplots comparing relationships between sampled pollutant concentrations and pollution indicators, by season, under temporal adjustment Equation 2. In both seasons, measured concentrations were inversely correlated with elevation (i.e., river valley); stronger correlations occurred during winter sampling (Spearman rho -0.42 to -0.72), and PM_2.5_ showed the weakest correlation with elevation, overall (summer rho -0.11, winter -0.42). Traffic density was positively correlated with NO_2_ concentrations, in both seasons (summer rho 0.33, winter 0.36). Emission-weighted proximity to industry, a highly left-skewed indicator, was not significantly correlated (i.e., p < 0.05) with measured pollution concentrations in either season. Under regional background temporal adjustment (Equation 1), correlation patterns are qualitatively similar, with notable exceptions: a) the inverse relationship between elevation and pollutant concentrations is attenuated by at least 10% across pollutants and seasons; b) traffic density is not significantly correlated (p > 0.05) with NO_2_ in either season (summer rho 0.01, winter 0.19); and c) summer PM_2.5_ is more strongly correlated with emission-weighted proximity to industry (rho 0.32, p = 0.05) (Additional file 1: Figures S1 and Additional file 2: Figure S2).

**Figure 6:**
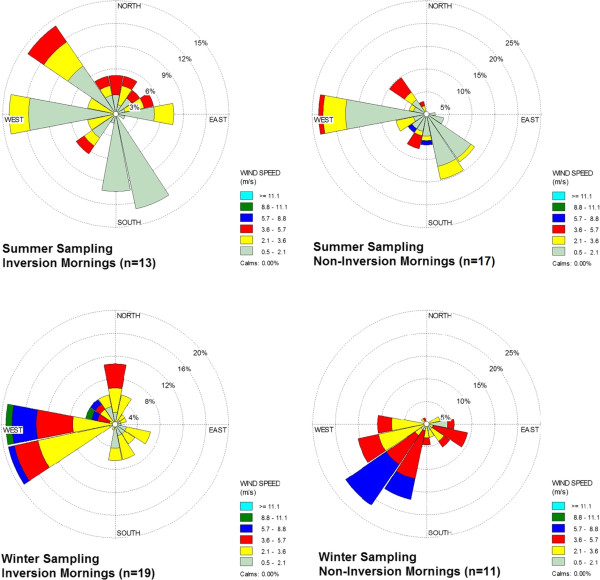
Wind direction and speed on inversion vs. non-inversion sampling mornings, by season.

Temperature inversion conditions were slightly more common during winter (2 to 4 mornings per 5-day sampling session) than summer sampling (1 to 3 mornings per session). Wind rose diagrams comparing inversion vs. non-inversion mornings show differing patterns in wind speed and direction (Figure 6). Across summer inversion mornings, wind directions were variable, with roughly 50% of all winds coming from either West, Northwest or South-Southeast directions, compared to predominantly Westerly winds on non-inversion mornings. Winds on winter inversion mornings were predominantly from the West and West-Southwest directions, while non-inversion morning winds were predominantly from the Southwest. Overall, wind speeds were generally higher during winter sampling sessions (winter mean = 3.20 m/sec, vs. summer mean = 1.79 m/sec), and sessions with lower average wind speeds (stratified at median) had higher concentrations of summer NO_2_ and winter BC (p < 0.05). Dominant wind directions were associated with winter PM_2.5_ (winds form South/Southwest) and winter NO_2_ (West/Northwest) concentrations, but not summer.

**Figure 7:**
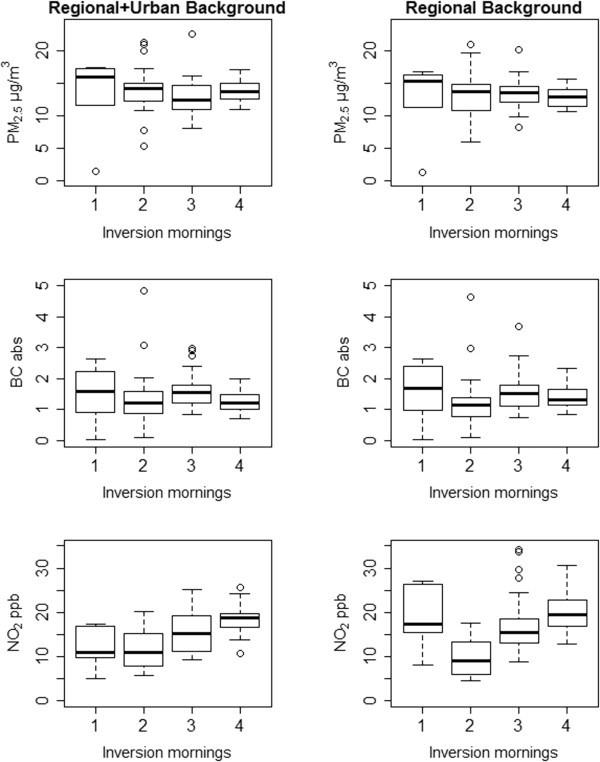
Pollutant concentrations by inversion frequency, by temporal adjustment method.

**Figure 8:**
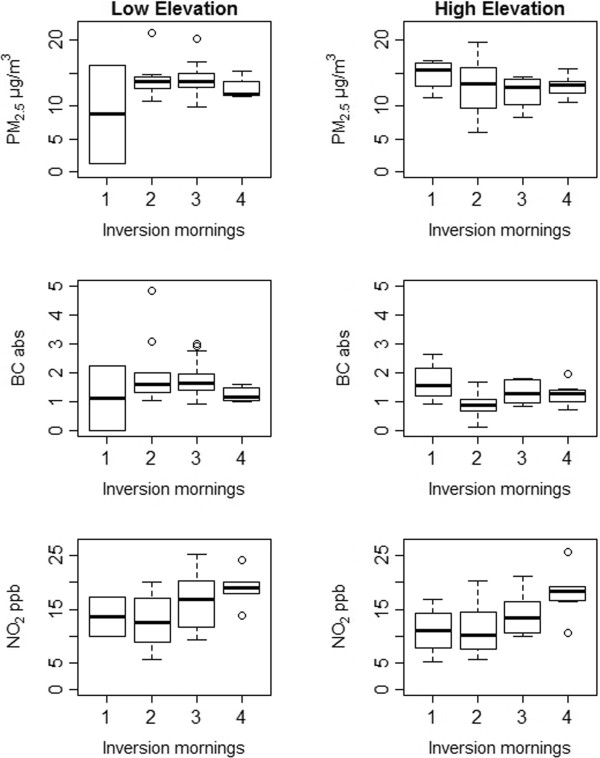
Pollutant concentrations by inversion frequency, among low- and high-elevation monitoring sites (n = 36 in each group).

Figure 7 compares the relationship between inversion frequency and pollutant concentrations, by temporal adjustment method, illustrating the importance of adjustment method for assessing the role of short-term meteorological events in spatial saturation studies. NO_2_ concentrations increased with number of inversion mornings per 5-day sampling session under the temporal adjustment method drawing information from both regional and local temporal trends (Equation 2), but not under regional-only adjustment (Equation 1). These relationships did not vary by season. This positive relationship between inversion frequency and NO_2_ was present among low- and high-elevation sites, however, with higher concentrations across low-elevation sights (Figure 8).

## Discussion

We present an approach for capturing intra-urban spatial contrasts in pollution concentrations, across complex terrain, during select hours of the day – here, to examine meteorological regimes. Our spatial saturation design captured source heterogeneity across our study domain, minimized spatial and temporal confounding within and across sampling sessions, and included topographic indicators to provide additional information on intra-urban variability in air quality. We offer an approach towards better understanding the impacts of short-term temperature inversions on spatial variation in air quality, by leveraging a programmable spatial monitoring system, diurnal variation in meteorology, and spatial gradients in topographic modifiers (i.e., elevation), and compare the efficacy of multiple temporal adjustment methods for this temporally-stratified spatial dataset.

This is the first study, to our knowledge, to use a spatial saturation approach to disentangle local pollution sources across the Pittsburgh region, with specific attention to complex terrain and atmospheric inversions. We observed seasonal differences in pollutant concentrations, and spatially-distributed sampling captured greater variability in pollutant concentrations than did County regulatory monitors. PM_2.5_ concentrations were higher during summer sampling, consistent with a previous central-site particle monitoring study in Pittsburgh [22], and NO_2_ concentrations were higher during winter sampling.

We examined two temporal adjustment methods for deriving seasonal mean concentration estimates at distributed locations, and found that the choice of reference site(s) influenced observed relationships between measured concentrations and emissions indicator strata (i.e., elevation, traffic density, industrial emissions) for NO_2_, but not for PM_2.5_ or BC. This discrepancy is likely because our upwind (regional) site, in a sparely-populated area, effectively captured background variation in non-reactive pollutants with upwind sources (such as PM_2.5_ or BC), but not highly photochemically reactive pollutants (such as NO_2_). Averaging the two reference monitors effectively captured some aspects of both the regional (i.e., long-range transport, meteorology) and local (i.e., topography, urban source activity variation) time-varying factors that influence intra-urban air quality. As such, we found that the appropriate background reference trend differs between pollutants, and determined that temporal adjustment using regional and urban background trends was more robust for a multi-pollutant study with a focus on potential meteorological effects on local concentrations.

While the pollution source indicators used to allocate sampling sites were consistent with the LUR literature, we did not find strong overall relationships between measured pollutants and traffic density or emissions-weighted proximity to industry. This is not entirely surprising, as the GIS-based indicators used for site selection and allocation were intentionally inclusive of multiple components of source activity. For example, we developed a multi-pollutant indicator of industrial emissions to prevent biasing study design toward one type of facility or chemical, and this underlying study design is well-suited for future LUR models that will use refined metrics (e.g., SO_2_ emissions in tons, stack height). Likewise, our traffic density indicator includes total vehicle counts on major and minor roads, but local traffic-related pollution may be driven by specific aspects of traffic patterns (e.g., average vehicle speed, idling) and fleet composition (e.g., diesel trucks, bus traffic).

An alternate explanation for weak overall source-concentration relationships are modifying effects of topography and meteorology. Across our distributed monitoring sites, elevation was inversely associated with BC and NO_2_ concentrations, in both seasons. Among low-elevation sites, where we hypothesized that inversion effects would be exacerbated, NO_2_ concentrations were higher during sampling sessions with 3 or more inversion mornings across low-elevation monitoring sites, but no statistically significant differences were found for PM_2.5_ or BC. This finding suggests that inversions have different effects across pollutants. This difference may by a function of local sources versus long-range pollution transport; if PM_2.5_ predominantly originates from long-range sources, it may demonstrate lesser local trapping in industrial valleys during inversion events, while the opposite may be true for BC and NO_2_, if they primarily arise from local sources. Lower regional wind speeds on inversion mornings also help explain different effects across pollutants, particularly during summer sampling; higher NO_2_ and BC during sampling sessions with multiple inversions may be attributable to less advection and dispersion of locally-generated pollutants, compared to long-range PM_2.5_. Other potential explanations for different observed inversion effects across pollutants, which are not mutually exclusive, include vertical emission location (i.e., industrial stacks versus on-road traffic), and atmospheric chemistry (i.e., reactivity and transformation rates) [44].

These findings are in keeping with other urban-scale (as opposed to simulation or regional-scale) monitoring studies of inversion effects on pollutants. Wallace et al. (2010) identified similar differential inversion effects by elevation in a mobile monitoring study in Hamilton, Ontario, but saw effects for both PM_2.5_ and NO_2_[45]. These inconsistent PM_2.5_ findings may be due to different topography-meteorology interactions in Hamilton (steep escarpment dividing the city into contiguous elevation zones), versus Pittsburgh (complex river valleys), or to different study designs; Wallace et al. took multiple observations at six Hamilton locations over three years under variable inversion conditions, while our study collected samples across 36 sites spatially distributed across a finer gradient of source-elevation profiles. In a separate fixed-site (n = 3) study of inversion effects on pollutants in Hamilton, Ontario, Wallace and Kanaroglou (2009) identified differential inversion effects on PM_2.5_ and NO_2_ by timing of inversion and season, and explained this difference by a range of meteorological factors, including prevailing wind directions [46].

We found some evidence that the impact of inversion conditions on the source-concentration relationship may vary by topography, pointing to complex challenges for integrating meteorological factors into saturation studies. Spatially, regional-scale meteorological data (e.g., atmospheric sounding, airport-measured wind direction) may not accurately capture intra-urban spatial variation of inversion dynamics. For example, the thermal profile of urban areas differs from relatively suburban areas, and katabatic cold air drainage from more densely built, warmer areas (i.e., urban heat island) can result in greater heating of clouds and inversion layer thickness, as compared to suburban areas [47,48]. Similarly, cool air pooling in river valleys may delay surface warming, and extend inversion conditions longer than reflected by sounding data. Temporally, integrated samples, though well-suited for deriving spatially-refined seasonal concentration estimated, are not ideal for interpreting effects of short-term meteorological events. Our approach examining inversion frequency within integrated sampling sessions was sensitive enough to detect some differences between inversion and non-inversion days, but not to examine how specific inversion characteristics (e.g., mixing height, vertical lapse rate) are implicated in inversion-related pollution effects. However, just as spatial saturation studies control for time-varying day-of-week and within-day variations in pollution source activity (e.g., rush hour) by design, this work, and others’ [45], supports conditioning site allocation on topographic modifiers of inversion effects (i.e., elevation) as a useful approach.

### Strengths

A unique strength of our study is the use of programmable monitors to synchronize active PM_2.5_ sampling across many locations, with a focus on inversion-prone morning rush hours. The primary strength and novelty of this work is the contribution to methods and metrics for understanding the role of topographic and meteorological factors on intra-urban air quality variability using a spatial saturation approach. Strengths of our study design include: monitoring across multiple seasons, measuring multiple pollutants, and spatially saturating a complex domain to systematically disentangle important local exposure factors. Key strengths of our instrumentation and data quality include: sampling near breathing zone height and excellent data quality and completeness. Our site allocation methodology minimized confounding of spatial and temporal factors, and provided useful information for refining and interpreting GIS-based pollution source indicators for LUR modeling.

### Limitations

Regional meteorological measures (e.g., wind speed and direction) limit our ability to assess differential effects on source-concentration relationships; integrated wind measurement instrumentation with each monitor, for example, would be ideal, but infeasible within allowed resources. Temporally, the potential effect of morning inversions on particle concentrations may have been diluted, if highest inversion-related pollution concentrations are found pre-sunrise, as our monitoring began at 6:00 AM, coinciding with morning rush hour. Additionally, low between-session variability in inversion frequency – all sessions in summer and winter had at least one inversion morning – limited examination of pollution effects; this observation also reinforced the importance of understanding this potentially important driver of regional air quality variability. Power calculations for spatial saturation designs vary by location, and 36 sampling locations may have been insufficient to saturate our domain, despite systematic allocation. Though previous LUR studies have cited 40 cites as a minimum for urban monitoring [41,42], and others have successfully used as few as 20 sites [49], one study in New York City demonstrated the utility of as many as 150 sites in a dense, spatially heterogeneous urban area [5,7]. To increase saturation for multi-year LUR modeling, we repeated this study design during a second year (summer and winter). Finally, due to monitoring solely during winter and summer months, we are unable to draw any conclusions about potential interaction of meteorology, topography and pollution during spring and fall seasons.

## Conclusion

In conclusion, we measured wide variability in multiple pollutants across the Pittsburgh metropolitan area, and found some evidence for modification of the inversion-concentration relationship by topography. This work contributes to methods for accurately capturing time-varying factors in spatial saturation studies by design, through targeting specific hours during which meteorological processes are hypothesized to have greatest impacts on local pollution concentrations, utilizing multiple reference monitors to understand spatial heterogeneity in temporal trends, and incorporating topographic gradients, which may interact with meteorological process, in sample allocation.

## Abbreviations

ACHD: Allegheny County Health Department; AADT: Annualized Average Daily Traffic; BC: Black carbon; GIS: Geographic Information Systems; LPM: liters per minute; LUR: Land use regression; NEI: National Emissions Inventory; NO_2_: Nitrogen dioxide; NO_X_: Nitrogen oxides; O_3_: Ozone; PA: Pennsylvania; PM_2.5_: particulate matter; SO_2_: Sulfur dioxide; VOC: Volatile organic compound.

## Competing interests

The authors declare that they have no competing interests.

## Authors’ contributions

JLCS and DM were primarily responsible for GIS-based analyses and study design. LC, LGC, SG, JH, DM, CR, ST, and BT carried out field implementation and aspects of study design. LC and BT carried out laboratory analyses. BT and KNS analyzed meteorological data. JLCS performed statistical analyses. JG and HE supported instrumentation. FH contributed to overall study design, and JEC oversaw all aspects of study design and implementation. All authors read and approved the final manuscript.

## Supplementary Material

Additional file 1: Figure S1 Summer PM_2.5_, BC and NO_2_ concentrations across source indicators and elevation (temporally adjusted using regional reference trend (Equation 1)). Click here for file

Additional file 2: Figure S2 Winter PM_2.5_, BC and NO_2_ concentrations across source indicators and elevation (temporally adjusted using regional reference trend (Equation 1)). Click here for file
