# Supplementary figures and images for: Saturation sampling for spatial variation in multiple air pollutants across an inversion-prone metropolitan area of complex terrain
Source: Environ Health. 2014 Apr 16;13:28. doi: 10.1186/1476-069X-13-28 (PMC4021317; doi:10.1186/1476-069X-13-28)

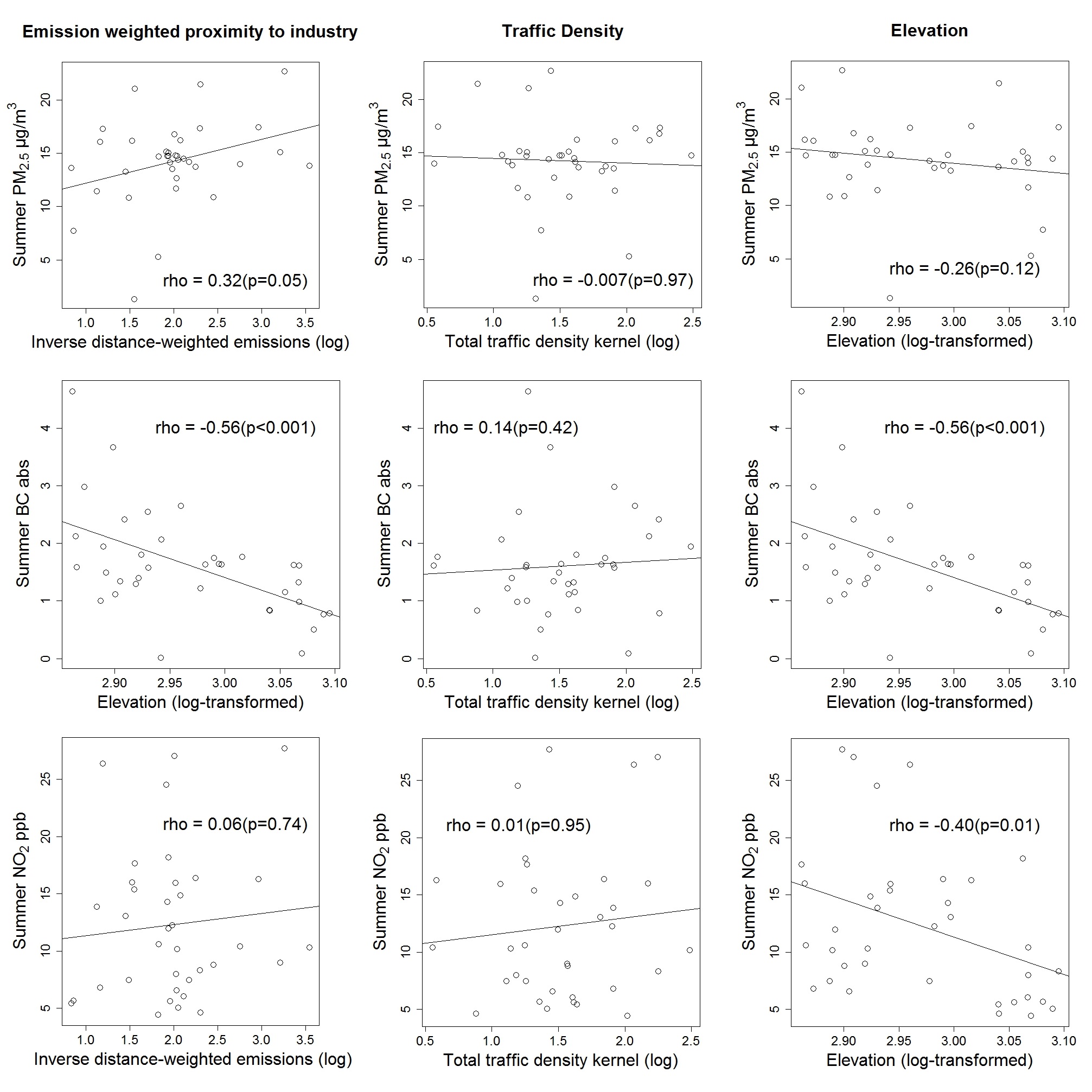

Supplement: Additional file 1: Figure S1 — Summer PM2.5, BC and NO2 concentrations across source indicators and elevation (temporally adjusted using regional reference trend (Equation 1)). [file 1476-069X-13-28-S1.jpeg]

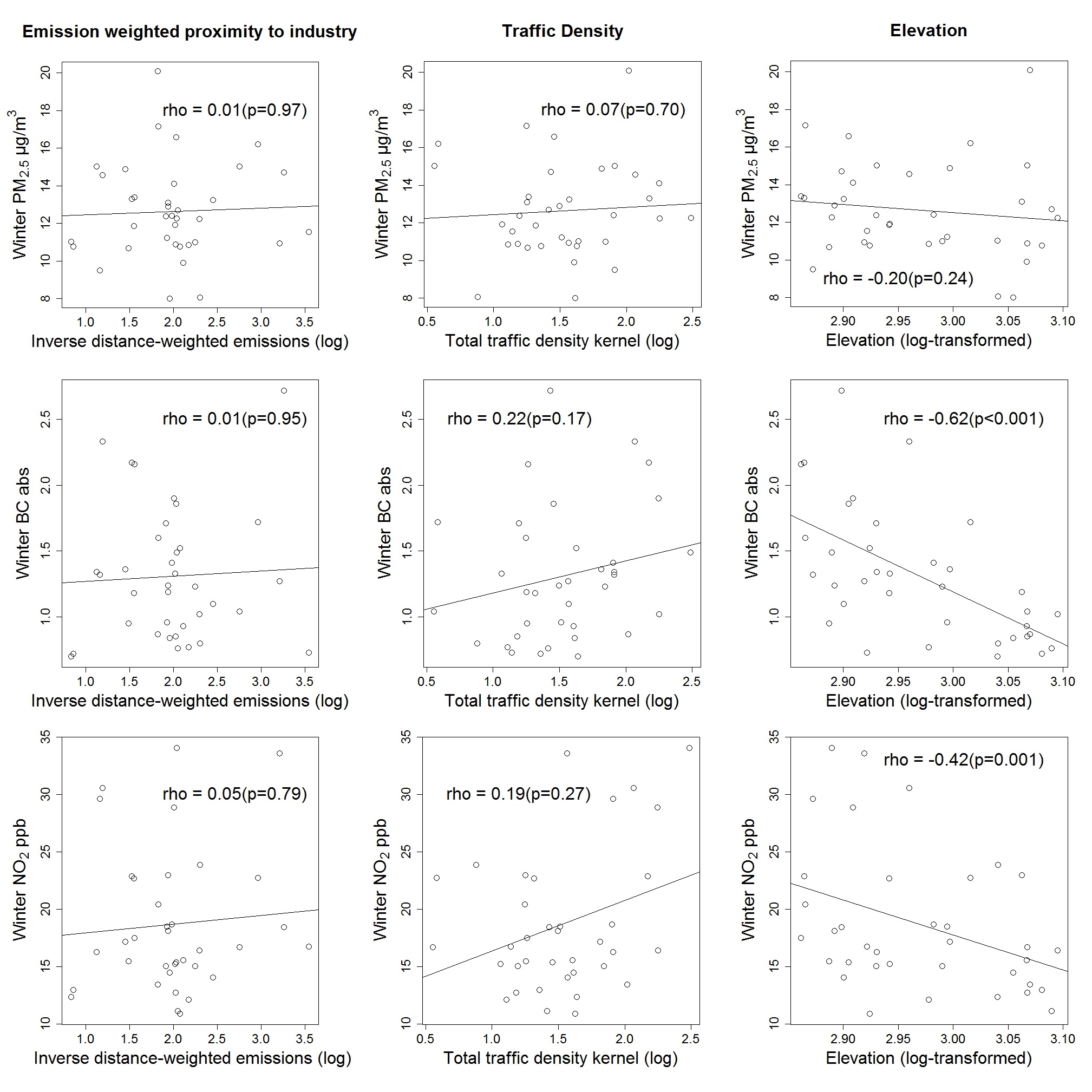

Supplement: Additional file 2: Figure S2 — Winter PM2.5, BC and NO2 concentrations across source indicators and elevation (temporally adjusted using regional reference trend (Equation 1)). [file 1476-069X-13-28-S2.jpeg]
